# Social determinants and cardiovascular disease mortality in Panama, 2012–2016

**DOI:** 10.1186/s12889-019-6508-8

**Published:** 2019-02-15

**Authors:** Luis Quiel, Ilais Moreno Velásquez, Beatriz Gómez, Jorge Motta, Víctor Herrera-Ballesteros

**Affiliations:** 1 Gorgas Memorial Institute for Health Studies, Panama City, Panama; 2 National Secretariat for Science and Technology, Panama City, Panama; 3 Department of Research and Health Technology Assessment,, Gorgas Memorial Institute for Health Studies, Panama City, Panama

**Keywords:** Social determinants, Cardiovascular mortality, Panama

## Abstract

**Background:**

The aims of this study were to determine the geographic and time variation of social determinants of health (SDH) and cardiovascular disease (CVD) mortality in Panama from 2012 to 2016, and to identify which of the SDH has the strongest correlation with a socioeconomic index (SEI).

**Methods:**

We conducted an ecological study obtaining mortality from the National Mortality Register and socioeconomic variables derived from the National Household Survey (NHS). The International Classification of Diseases 10th revision codes I20–I25 and I60–I69 were used for ischemic heart disease (IHD) and stroke, respectively. Standardized age-adjusted mortality rates were calculated by direct method. Mortality rates and socioeconomic variables were evaluated together in a panel data model. A SEI was developed from factorial analysis by principal components with a polychoric correlation matrix. Provinces and regions were categorized in tertiles according to median value of the SEI score.

**Results:**

The NHS evaluated an average of 15,919 households per year. The mean of age throughout the study period was 41 years. The average monthly income increased, from US$ (SD) 331.94 (5.38) in 2012, to 406.24 (5.81) in 2016, whereas the social security health coverage remained in a range of 57–58%. The mean number of school years was twelve. Significant geographical and temporal variations in social determinants and mortality rates were observed throughout the country. Colon, categorized in the middle tertile according to the SEI, presented higher IHD mortality rates. Darién (in the lowest SEI tertile) Colón and Herrera had higher stroke mortality rates. The SEI categorized indigenous territories in the lowest tertile. Total years of education was the strongest correlated variable with the SEI, when we excluded the population living in indigenous territories. However, when this population was included, social security coverage had the strongest correlation with the SEI.

**Conclusion:**

We observed geographical and temporal disparities in SDH and CVD mortality rates. Further epidemiological studies are warranted in the provinces of Colón, Darien, Herrera and Los Santos to explore in-depth the higher CVD mortality rates observed in these provinces.

**Electronic supplementary material:**

The online version of this article (10.1186/s12889-019-6508-8) contains supplementary material, which is available to authorized users.

## Background

Cardiovascular disease (CVD) claimed 17.7 million lives in 2015, and this represented approximately 31% of all global deaths [1]. From 2006 to 2016, despite efforts in prevention, screening and treatment, CVD remains the main cause of death in most countries [2, 3]. Nevertheless, in this same period of time, global death rates from CVD decreased by 14.5% [2]. In 2016, 85.1% of all CVD deaths were produced by ischemic heart disease (IHD) and stroke combined [2].

Recently, a multi cohort study and meta-analysis showed that a low socioeconomic status had a comparable effect in individual’s health than the traditional risk factors [4]. These findings suggest that socioeconomic adversity should be considered as a modifiable risk factor in local and global strategies directed at reducing premature mortality. Studies conducted in European countries have consistently demonstrated the contribution of social inequalities to CVD incidence and mortality [5, 6].

In Latin America (LA), CVD is responsible for 33.7% of total mortality [7]. In the last decade, important social and economic changes have occurred in this region, however, according to the World Economic Forum and the International Monetary Fund, LA remains one of the most unequal region worldwide [8]. To the best of our knowledge, studies that explore the social determinants of health in CVD incidence and mortality are scarce in Central America.

Panama is an upper-middle-income country located in Central America with a population of > 4 million. The country is politically divided in ten provinces (Bocas del Toro, Coclé, Colón, Chiriquí, Darién, Herrera, Los Santos, Panama, Veraguas and West Panama) and five indigenous territories (Emberá-Wounaan, Guna Yala, Ngöbe-Buglé, Kuna de Madugandí and Kuna de Wargandí – the first three are categorized as provinces). According to the latest estimates, indigenous peoples represent 12% of the total population in the country [9]. CVD mortality trends have declined in recent years [10], yet CVD is still the leading cause of death, responsible for 28% of national mortality [11].

Even though Panama has been one of the fastest growing economies in the world in the last 10 years, socioeconomic inequalities persist, especially in indigenous territories. The World Bank estimated a Gini index of 50.8 in 2015, ranking Panama as a highly unequal country [12]. Results from the Multidimensional Poverty Index (MPI) indicated that 19.1% of the Panamanian population live in a multidimensional poor household [13]. Furthermore there is increasing evidence of the association between social determinants of health (SDH) and non-communicable diseases in Panama. Observational studies have shown that the lack of social security coverage in patients with gastric cancer is an independent risk factor for all-cause mortality; monthly family income has an inverse association with chronic kidney disease and monthly individual income less than US$ 100 has a strongest correlation to mortality due to diabetes mellitus (DM) [14–16]. However, the SDH in relation to CVD mortality in Panama remains to be explored.

The aims of this study were to: (i) determine the geographic and time variation of SDH and CVD mortality and (ii) identify which of the social determinants has the strongest correlation with a socioeconomic status index.

## Methods

We designed an ecological study in an attempt to determine if there was any relation between SDH (defined as years of education, social security coverage and monthly income) and mortality due to CVD in Panama. The data were obtained from the household survey (HS) and the National Mortality Register (NMR); both derived from the National Institute of Statistics and Census (INEC, from its Spanish translation Instituto Nacional de Estadística y Censo).

Data were analyzed at the provincial level except for the province of Panama, which was divided into four regions. In total, we studied eight provinces (Bocas del Toro, Coclé, Colón, Chiriquí, Darién, Herrera, Los Santos and Veraguas) and four regions (Panama, San Miguelito, West Panama, East Panama).

### National Mortality Register

We conducted a registry-based analysis of mortality due to CVD, from the years 2012 to 2016. Several studies evaluated the data obtained from the Panamanian civil registration and vital statistics and classified it as high quality [17, 18]. CVD was defined and coded, following the tenth revision of the International Classification of Diseases (ICD-10). ICD-10 codes I20-I25 and I60-I69 were utilized for IHD and stroke, respectively.

### Household survey

The HS is a tool used to identify the employment status and socioeconomic conditions of the general population. It is widely used by governments to measure the characteristics of the working market and report unemployment. The World Bank, The International Labour Organization and the Oxford Poverty and Human Development Initative use the HS data to measure inequality calculate unemployment trends and construct the MPI, respectively. Other studies concerning reproductive, maternal, newborn, and child health have used HS as the primary data source to evaluate coverage indicators [19, 20].

In Panama, the HS has been applied biannually since 1963 (March and August) [21] . Households are randomly selected from the census performed in the country with the aim to be representative by provinces. Households are defined as the location used by a family or a group of people, with or without bonds, which share a unit or a person that lives alone.

The data is collected through person-in-person interviews, including all the members of the household at the moment of the interview and the questions are structured to correspond to the information of the week prior the interview. We analyzed all the data of surveys within each household, and thus, number of study participants may vary per year. Annually, 25% of households are replaced whereas 75% of households are revisited 4 times before they are replaced. In order to capture part of the economically active population, the reference period is extended to 6 months explaining why the survey is applied biannually. In Panama, March is a summer month where labor market widens, primarily because of seasonal employment and the beginning of the school year. Therefore, in the present study, we utilized the data obtained in August for the time frame 2012–2016, as March may not represent the market behavior compared to other months. An average of 15,919 households was surveyed per year. Since we aimed to address the economically active population, study participants below 15 years of age were excluded (*n* = 64,202)*.* Study participants with missing data for monthly income (*n* = 73) and years of education (*n* = 122) were further excluded. Thus, a total of *n* = 154,019 study participants were enrolled in the present study.

The data utilized for the present study is in the public domain and did not contain sensitive information (anonymous secondary data), consequently no ethics approval was required. Permission to have access to the variables utilized from the Household survey was obtained from the National Comptroller (Contraloría General de la República), head of the National Institute of Statistics and Census*.*

### Social determinants of health variables

Monthly income was analyzed as a continuous variable and defined as the total income an individual received per month in US dollar ($), including monetary and species earnings, subsidies and grants (the national currency is the Panamanian balboa, however it is pegged with the US dollar at par). It included primary income and the income earned with other economic activities. Social security coverage was categorized as the access to public social security health services, represented as contributor (active worker), beneficiary, retired, pensioner (permanent disability), retired or pensioner from another country and with no coverage. We dichotomized the variable as having or not social security coverage. Years of education were explored as a continuous variable, specifying the maximum number of years of education reached. The education in Panama is characterized in three levels: basic general education, middle education and higher education. Basic general education is compulsory and consists of a total of 11 years, including preschool, elementary and middle school. Middle education consists of an additional 3 years and is diversified in general, pedagogical and technical education. Higher education is subdivided in university and non-university education. Special education is imparted to children with limiting mental or physical conditions.

### Statistical analysis

Dichotomic variables were expressed as percentages and quantitative variables as mean and standard deviations (SD). Estimates were weighted to represent the total population of Panama per province and regions. The expansion factor was calculated and provided by the INEC.

Specific mortality rates for IHD and stroke were calculated separately for each year, province and sex, adjusted by age, based on the 2010 national census estimates and projections of the population per province and region. These mortality rates were standardized by direct method, using the World Health Organization (WHO) standard population. The groups were categorized in 5-year age groups; however, we reported specific mortality rates from the population aged 35 to 79 years.

To determine if there were changes throughout the years (within variation) and provinces/regions (between variation), the mortality rates and socioeconomic variables were evaluated together in an econometric analysis based on panel data models. The analysis was performed separately for IHD and stroke and by sex. Due to a high underreporting in mortality data, indigenous territories were not included in the panel data analysis [22]. The data was described, summarized and sorted for data panel analysis. The Durbin–Wu–Hausman test was applied to choose between the fixed and random effect models. The Breusch Pagan Lagrange Multiplier test was implemented to choose between random effect and pooled models. The results of this test suggested that a pooled model regression was not suitable. Moreover, within and between variations in standard deviation were found for IHD and stroke.

Because of these findings, socioeconomic variables were merged in a polychoric correlation matrix in order to perform a factor analysis by principal components. Three factors were introduced in the analysis. The common factor was extracted by the principal-component method and then an orthogonal varimax rotation was done in order to distribute the factor loadings. A Kaiser-Meyer-Olkin test was performed to measure how suitable the data was for factor analysis. This factor was predicted for each value and then a SEI was obtained per province/region. Provinces and regions were categorized in tertiles according to the value of its SEI score median.

Analysis were performed in Microsoft Excel 2013 and in Stata IC version 14.0.

## Results

Table 1 summarizes the baseline characteristics of the HS and the CVD mortality rates in the study period. The mean of age throughout the years of study was 41 years. The monthly income increased, from US$ (SD) 331.94 (5.38) in 2012, to 406.24 (5.81) in 2016 whereas the social security coverage remained in a range of 57–58%. The mean years of education in the study population was twelve years. After exclusion of surveys from indigenous territories, we observed higher percentages of social security coverage and monthly income. Overall, higher mortality rates for IHD (63.8 per 100,000) and stroke (47.1 per 100,000) were observed in 2013 (Table 1).

**Table 1:** Distribution of baseline characteristics in the Household Survey and Cardiovascular Disease mortality rates in Panama, 2012–2016

	2012	2013	2014	2015	2016
Household Survey					
Households surveyed	16,143	16,143	16,658	15,326	15,326
Total of surveys	45,636	44,237	43,719	42,396	42,233
Study population ≥ 15 years	31,586	30,885	30,774	30,324	30,450
Weighted population ≥ 15 years	2,659,822	2,719,844	2,782,076	2,846,612	2,909,973
Age, mean (SD)	40.4 (0.11)	40.6 (0.13)	40.8 (0.12)	41.1 (0.13)	41.0 (0.13)
Monthly income, mean (SD)	331.9 (5.38)	349.8 (6.36)	364.6 (5.45)	397.9 (7.33)	406.3 (5.81)
Access to social security (%)	58	57	56	57	57
Years of education, mean (SD)	11.9 (0.03)	11.9 (0.36)	11.9 (0.03)	12.1 (0.03)	12.2 (0.04)
Study population ≥ 15 years excluding indigenous territories	29,450	28,917	28,878	28,516	28,649
Weighted population ≥ 15 years excluding indigenous territories	2,530,350	2,589,545	2,650,919	2,714,565	2,777,312
Age, mean (SD)	40.6 (0.12)	40.7 (0.13)	41.0 (0.13)	41.3 (0.13)	41.2 (0.13)
Monthly income, mean (SD)	346.3 (5.63)	365.5 (6.65)	380.6 (5.69)	414.4 (7.66)	422.6 (6.06)
Access to social security (%)	61	60	59	59	59
Years of education, mean (SD)	12.2 (0.03)	12.2 (0.04)	12.3 (0.03)	12.4 (0.03)	12.5 (0.04)
National Mortality Register					
Mortality rate IHD (× 100,000 inhabitants)	62.8	63.8	62.6	51.1	48.2
Mortality rate stroke (× 100,000 inhabitants)	45.7	47.1	46.8	44.2	46.7

Missing data: monthly income (*n* = 73), years of education (*n* = 122)

### National Mortality Register

**Fig. 1:**
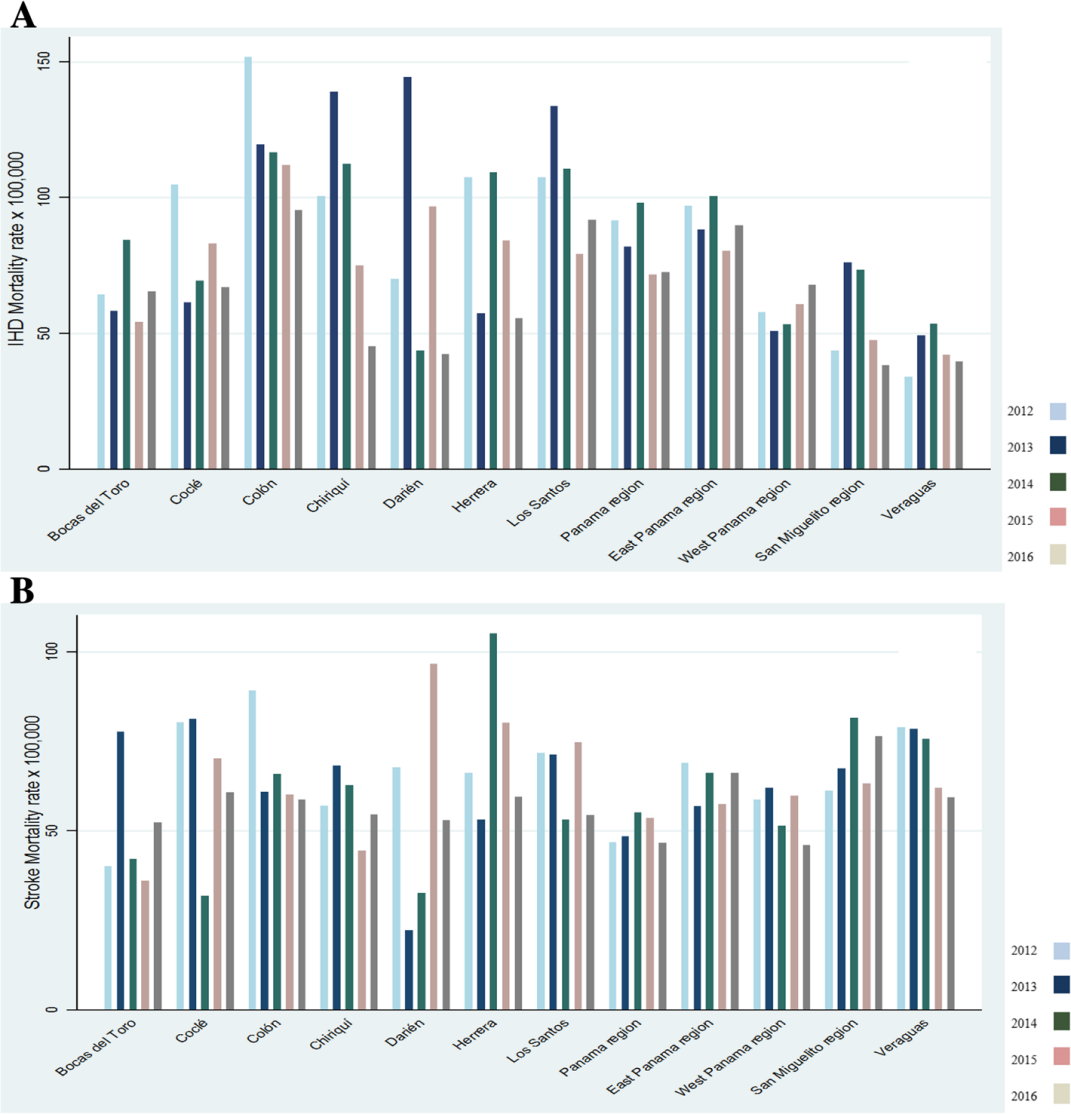
Age-adjusted ischaemic heart disease (**a**) and stroke (**b**) mortality rates in men per province and region in Panama, 2012–2016

**Fig. 2:**
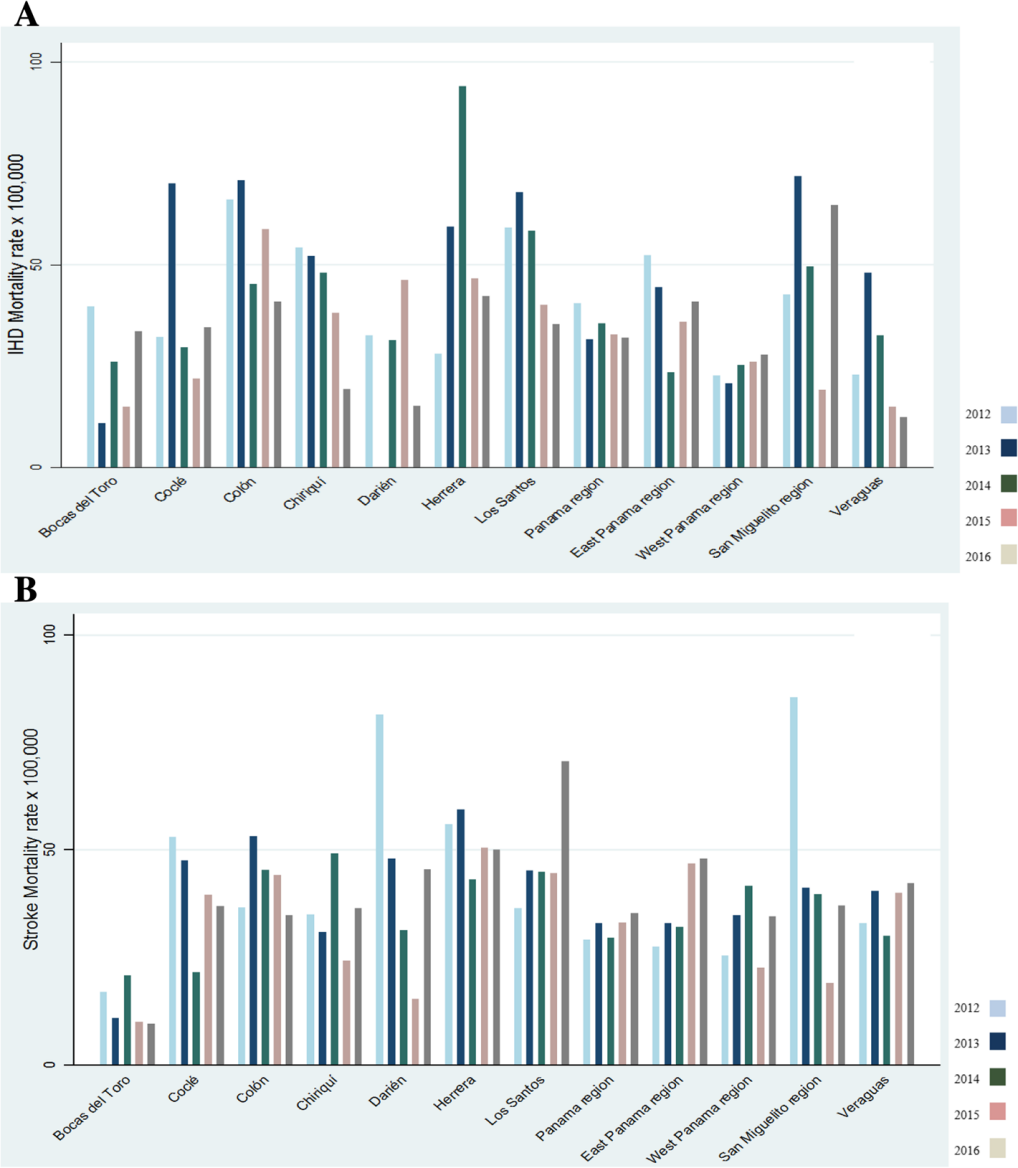
Age-adjusted ischaemic heart disease (**a**) and stroke (**b**) mortality rates in women per province and region in Panama, 2012–2016

From 2012 to 2016, 15,661 deaths were attributed to CVD (IHD *n* = 8144 and stroke *n* = 7517). As shown in Fig. 1 (men), standardized and age-adjusted specific mortality rates (× 100,000 inhabitants) for IHD (Panel A) were higher in Colón for all the years, [2012 (151.9), 2014 (116.8), 2015 (112.1), 2016 (95.3)], except in 2013 (Darién 144.3). Likewise, in women (Fig. 2, Panel A), Colon presented the highest IHD mortality rates 66.2 and 58.8 (× 100,000 inhabitants) in 2012 and 2015, respectively. The region of San Miguelito had the highest IHD mortality rates in women (71.9 and 64.7) in 2013 and 2016.

In men, the provinces with the highest mortality rates (× 100,000) due to stroke (Fig. 1, Panel B) were Colón (89.4 in 2012), Coclé (81.3 in 2013), Herrera (105.2 in 2014), Darién (96.7 in 2015) and the region of San Miguelito (76.5 in 2016). In women, compared to other provinces (Fig. 2, Panel B), Herrera presented higher stroke mortality rates in 2013 (59.4) and 2015 (50.5), whereas Los Santos had the highest mortality rate in 2016 (70.6).

### Household survey

Additional file 1 presents the baseline characteristics of socioeconomic determinants in the HS per region and year. Compared to other regions, the indigenous territories and Darién had the most limited access to social security (4–15% and 17–22%, respectively), followed by East Panama region (32–42%). Panama region and San Miguelito region had the highest monthly incomes whereas the population of Darién (except for 2014) had the lowest mean of monthly income (below US$215.00).

Only the study population of the province of Panama (Panama, San Miguelito and West Panama) and the province of Colón achieved the compulsory years of education in the study period (mean). Overall, years of education were higher in women compared to men in all the regions for all the years (except for indigenous territories); however, average monthly income was higher in men doubling women’s monthly income.

The data panel analysis assessed temporal and geographical changes. As shown in Additional file 2, there were higher variations in all the socioeconomic variables and in IHD mortality in men throughout the provinces and regions (between). Similarly, higher variations were observed in IHD mortality in women and stroke mortality throughout the years (within variation).

**Fig. 3:**
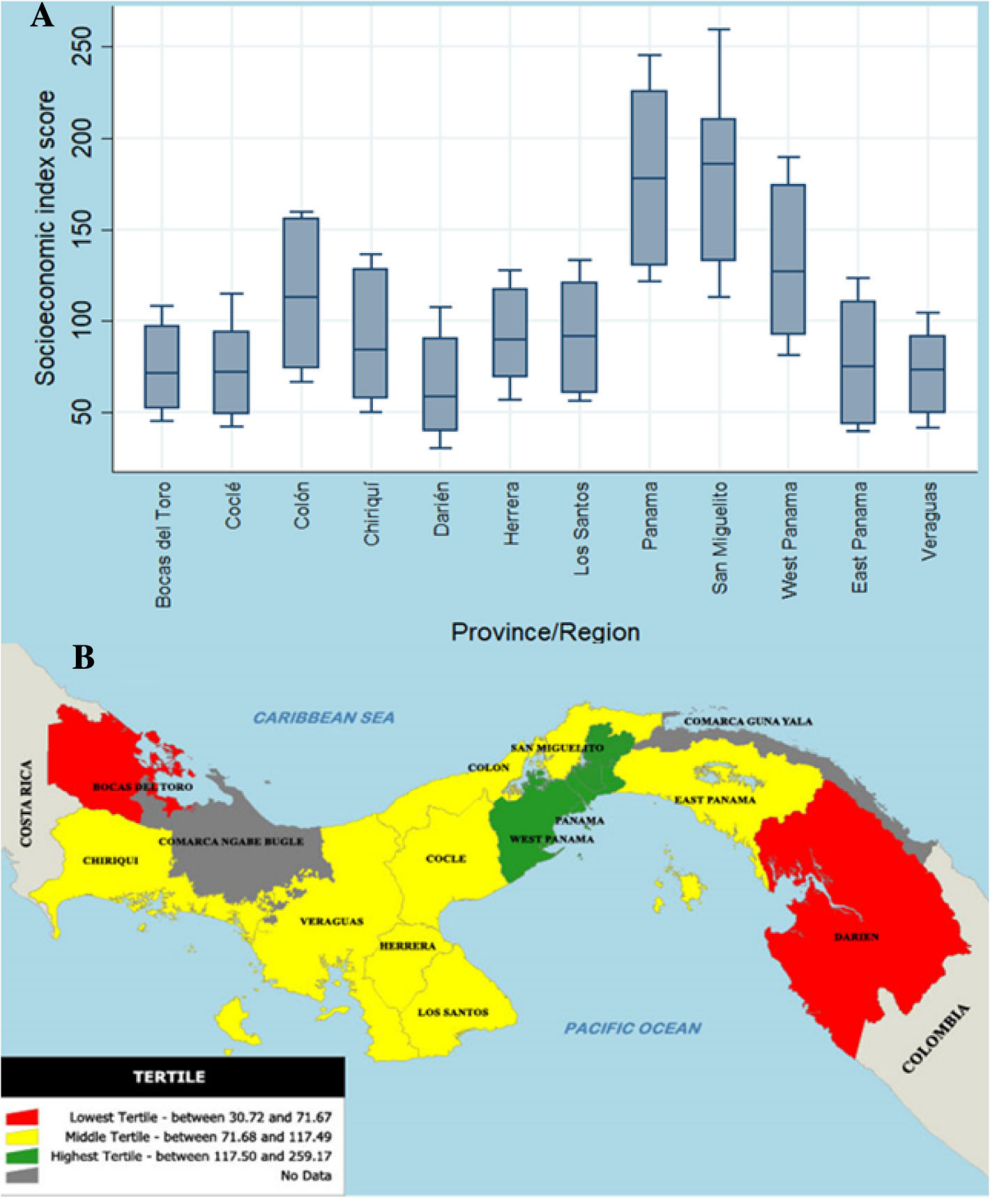
Socioeconomic index score distribution per province and region in Panama. Map constructed at the Gorgas Memorial Institute using the softwares Manifold version 8.0.5 and Qgis version 2.8. Maps were developed using geographical coordinates from the National Institute of Statistics and Census (INEC, from its Spanish translation Instituto Nacional de Estadística y Censo). Panel **a** shows the SEI score ranking per province and region. Panel **b** shows the geographical distribution according to the SEI score

Fig. 3 (Panel A) shows the SEI score ranking per province and region. The regions of San Miguelito, Panama and West Panama ranked as first, second and third, respectively, with the highest SEI scores. Darien ranked last, preceded by Bocas del Toro. Panel B shows the geographical distribution according to the SEI score. SEI scores were categorized into tertiles. The rationale behind this division was to observe if provinces with similar socioeconomic index had similar patterns with respect to the cardiovascular mortality rates. The lowest tertile was composed by Darién and Bocas del Toro. The middle tertile had Coclé, Veraguas, East Panama region, Chiriquí, Herrera, Los Santos and Colón whereas the highest tertile consisted of West Panama region, Panama region and San Miguelito region. When integrating the indigenous territories in the SEI, we observed that Emberá-Wounaan, Guna Yala and Ngöbe-Buglé were included in the lowest tertile. Darién and Bocas del Toro were recategorized to the middle tertile, while Colón was re-categorized to the highest tertile

Factorial analysis extracted the common factor (factor 1). This factor had the highest proportion (0.6044) compared to the other factors and therefore it was utilized. As shown in Table 2, the variable with the strongest correlation to factor 1 after rotation of factor loadings was years of education (0.9205), followed by social security access (0.8865). The overall Kaiser-Meyer-Olkin was 0.643, an acceptable value that indicates the correlation matrix is appropriate for factor analysis [23].

**Table 2:** Score coefficients extracted by principal-components method with ortogonal varimax rotation

Variables	Factor 1	Kaiser-Meyer-Olkin
Years of education	0.9205	0.5996
Social security access	0.8865	0.5941
Monthly income	0.7728	0.8644
Province/region	0.4328	0.6300
Overall		0.6430

## Discussion

We evaluated CVD mortality rates and SDH in eight provinces and four regions throughout 2012 to 2016. We observed geographical disparities in the SDH evaluated and in IHD mortality rates in men within the country. Temporal differences were seen in IHD mortality rates in women and stroke mortality rates. Furthermore, years of education and social security presented the strongest correlation with the SEI.

### Geographical and temporal variations in SEI and CVD mortality

The constituent elements of our SEI index: income, education, and access to social security are key factors associated with health by being wholly or partially classed as social determinants [24–27]. Our results indicated regional disparities in SDH within the country, yet disparities between years were stable. Education, one of the most important determinants of health, presented the highest burden in our SEI index. In Panama, the current government expenditure on education represents 3.2% of the GDP, a low percentage compared to neighboring countries like Costa Rica (7.06%), Cuba (12.83%) and Chile (4.90%) [28]. According to data from the World Bank, the government expenditure was 4.04% in the 1970s [28].

Although the average years of education was higher in women, the monthly income in men doubles the one of women. Similarly, a previous study using data from the HS concluded that men had an income 28% higher than women, despite men had higher rates of incomplete elementary and high school education [29]. In the context of these findings, gender-related policies need to be investigated-in depth. We found that social security coverage was 57–58% at a national level, a lower percentage than that reported by the INEC (an average of 75.5% from 2012 to 2016) [30]. This discrepancy might be explained because during the HS, participants were asked if they have social security coverage the week prior the survey. In addition, there has been an increase in informal employment in the country, with 40.2% of the employed population in informal employment in 2016 [31]. Remarkably, the socioeconomic variable with the strongest correlation with the SEI was social security coverage when including the indigenous population. In agreement with our results, the MPI reported that in almost all indigenous territories, more than 90% of the population was multidimensional poor, followed by Bocas del Toro (45%) and, Darién (40%) [13].

Our results suggest that there was a decrease in mortality rates in IHD and stroke during the study period. Similarly, a recent study reported a decline in the trend of stroke mortality from 2001 to 2014 and a decrease in IHD mortality that started in 2010 [10]. Whether the decrease in trends occurred similarly in provinces and regions is less clear. Overall, we observed that mortality rates varied through years and provinces, especially mortality related to IHD. This variation could be explained, in part, by certifications of the cause of death, higher coverage of ambulance services since 2007 and greater availability of health services [32] . Less variation in mortality rates was found in the regions of Panama, West Panama and San Miguelito, possibly due to the proximity to tertiary care hospitals and better death registration and certification.

### Social determinants and IHD mortality

For most of the years studied, Colon presented higher mortality rates for IHD in both women and men. Similarly, the highest mortality rate due to Diabetes Mellitus (DM) had been documented in this province [16]. This is despite the fact that Colon SEI score ranks above the middle tertile. Previous findings from a population-based study performed in Panama and Colon reported elevated prevalence of high blood pressure (HBP) among individuals with elementary education and illiteracy, compared to those with higher levels of education [33]. Colon, located 80 km from the capital city, is the third most populated province in the country with the largest African descent population [34]. Colon has a Free Trade Zone, the second largest in the world, several important maritime ports and part of the Panama Canal. GDP is the second in the country, superposed only by the province of Panama [35].

Colón has 74 health centers (1 secondary care hospital and 73 primary health care centers) [36] . Notably, more than half of the population of Colon had access to social security and therefore, it could be speculated that multiple factors may be playing an important role in the mortality rates observed in this province. Among the potential explanations, access and adherence to treatment deserves mention. In Colon, studies have reported that 65.6% of the population diagnosed with HBP were under antihypertensive treatment [33] and 72.2% of Colón’s population cannot afford out-of pocket expenditure in the private sector of medications [37]. In agreement with our findings, higher CVD mortality rates have been reported in municipalities that are closer to the US-Mexico border, despite having superior infrastructure and proximity to health care [25], probably explained by US acculturation, unequal access to healthcare system or specific factors in the Mexican population living in the border area [25]. Further, ethnicity, as a proxy for biological characteristics and cultural norms has been associated with increase in cardiovascular risk factors [38].

### Social determinants and stroke mortality

Los Santos and Herrera, with a known aging population, ranked in the middle tertile, and presented higher mortality rates for stroke, particularly in women. In line with our results, a study has reported higher self-reported prevalence of hypercholesterolemia, HBP, DM, and overweight in these provinces, but lower socioeconomic determinants [16].

Darién (with higher mortality rates for stroke in 2015 in men), was categorized in the lowest tertile of the SEI. Remarkably, Darién (along with indigenous territories) had lower amount of years of education (mean of 7 years), compared to the rest of the country. Mortality including premature mortality, increased with decreasing educational level and with increasing number of underlying diseases [24]. Similarly, there is evidence that socioeconomic risk factors may have a significant impact on stroke and mortality [26, 39]. Darien (located at the eastern end of the country-bordering Colombia) is an admixed population isolated by urban developments and limited of health public services. As of the year 2016, Darien had health centers from the MoH, yet no hospitals. Noteworthy, in the mortality context, deaths are underreported in Darien by 52%, therefore results must be interpreted with caution [28]. Interestingly, San Miguelito, a region with a high SEI index, albeit a high population density presented the highest stroke mortality rates in some years.

Several limitations must be addressed. First, we used socioeconomic variables that did not correspond to the individual’s mortality data, so causality must not be inferred and the present findings must be interpreted with caution, in order to avoid an ecological fallacy. Second, to avoid bias, we excluded indigenous territories from data panel analysis. Of note, the NMR underreporting is still an important limitation in indigenous territories. In the HS, socioeconomic variables were obtained from indigenous territories, however, because of social inequality; socioeconomic determinants in the indigenous territories differed importantly compared to other regions. When integrating this data in the factorial analysis and obtaining the SEI, the other regions had an overestimated SEI, limiting the comparisons in these regions. Finally, only five years were considered in the study because since 2012, the HS had a mayor variation in the sampling, and thus trend analysis was not applicable. However, to the best of our knowledge, this is the first study that attempts to explore CVD mortality in relation to socioeconomic determinants per province and region in Panama.

## Conclusion

We observed geographical and temporal disparities in socioeconomic determinants and CVD mortality rates. Total years of education had the strongest correlation with the (SEI), if we excluded the indigenous territories. However, if we included the indigenous territories, social security coverage had the strongest correlation with the SEI demonstrating the importance of access to health care. Further epidemiological studies aimed to disentangle the role of risk factors for IHD mortality in Colón are warranted. As for Los Santos, Herrera, San Miguelito and Darien, SDH in relation to mortality deserve to be investigated in-depth.

Panama has a multiethnic population with cultural diversity per region, a key aspect to consider when developing strategies and interventions to reduce health inequalities. In this perspective, political interventions with a multisectorial approach must be achieved in order to decrease the uneven distribution of SDH throughout the country. Additional, our study supports the need of validated socioeconomic data and the notion of an integrated register health system.

## Additional files

Additional file 1: Baseline characteristics of the household survey per province and region based on expanded data. The table shows the distribution of age mean, social security coverage, income mean and years of education mean by provinces and years analyzed in the present study. (DOCX 58 kb)

Additional file 2: Variation in socioeconomic variables and cardiovascular disease mortality rates. The table shows the variations in all the socioeconomic variables and IHD/stroke mortality in men and women throughout the provinces and regions (between) and throughout the years (within variation). (DOCX 16 kb)

## Abbreviations

CVD: Cardiovascular disease
DM: Diabetes Mellitus
GDP: Gross domestic product
HBP: High blood pressure
HS: Household survey
ICD-10: International Classification of Diseases tenth revision
IHD: Ischaemic heart disease
INEC: National Institute of Statistics and Census, from its Spanish translation Instituto Nacional de Estadística y Censo)
LA: Latin America
MPI: Multidimensional Poverty Index
NMR: National Mortality Register
OECD: Organization for Economic Co-operation and Development
PISA: Programme for International Student Assessment
PREFREC: Survey on Risk Factors Associated With Cardiovascular Disease, from its Spanish translation Prevalencia de Factores de Riesgo de Enfermedad Cardiovascular)
SD: Standard deviation
SDH: Social Determinants of Health
SEI: Socioeconomic index
TERCE: Third Regional Comparative and Explanatory Study
UNESCO: United Nations Educational, Scientific and Cultural Organization
WHO: World Health Organization
