# Supplementary material for: Social determinants and cardiovascular disease mortality in Panama, 2012–2016
Source: BMC Public Health. 2019 Feb 15;19:199. doi: 10.1186/s12889-019-6508-8 (PMC6377740; doi:10.1186/s12889-019-6508-8)
Supplement: Supplementary file 1 — Baseline characteristics of the household survey per province and region based on expanded data. The table shows the distribution of age mean, social security coverage, income mean and years of education mean by provinces and years analyzed in the present study. (DOCX 58 kb) [file 12889_2019_6508_MOESM1_ESM.docx]

**Additional file 1. Baseline characteristics of the household survey per province and region based on expanded data**

| **2012** | | | | | | |
| --- | --- | --- | --- | --- | --- | --- |
| **Province/Region** | **n =** | **Sex** | **Age mean (SD)** | **With SS coverage** | **Income mean (SD)** | **Years of education mean (SD)** |
| **Bocas del Toro** | **2,094** | M | 36.4 (0.49) | 58% | 266.96 (12.34) | 9.62 (.1408) |
|  | **2,065** | F | 34.7 (0.46) | 50% | 132.95 (9.31) | 9.81 (.1642) |
|  | **4,159** | T | 35.6 (0.34) | 54% | 199.38 (7.82) | 9.72 (.1083) |
| **Coclé** | **1,576** | M | 41.3 (0.59) | 50% | 252.11 (11.51) | 10.17 (.1280) |
|  | **1,532** | F | 41.3 (0.59) | 56% | 117.83 (7.44) | 10.98 (.1492) |
|  | **3,108** | T | 41.3 (0.42) | 53% | 184.69 (6.99) | 10.58 (.0987) |
| **Colón** | **1,598** | M | 37.6 (0.53) | 64% | 448.69 (18.45) | 11.99 (.1277) |
|  | **1,649** | F | 39.4 (0.53) | 58% | 203.79 (11.65) | 12.51 (.1354) |
|  | **3,247** | T | 38.5 (0.37) | 61% | 322.18 (11.09) | 12.26 (.0934) |
| **Chiriquí** | **1,725** | M | 41.6 (0.55) | 51% | 289.72 (10.25) | 10.98 (.1416) |
|  | **1,831** | F | 42.1 (0.53) | 59% | 140.19 (8.36) | 11.93 (.1427) |
|  | **3,556** | T | 41.9 (0.38) | 55% | 211.76 (6.73) | 11.48 (.1011) |
| **Darién** | **1,215** | M | 39.8 (0.68) | 20% | 222.99 (13.32) | 8.08 (.1633) |
|  | **1,102** | F | 37.1 (0.67) | 24% | 82.42 (7.62) | 8.92 (.2061) |
|  | **2,317** | T | 38.5 (0.48) | 22% | 154.91 (8.03) | 8.49 (.1311) |
| **Herrera** | **1,328** | M | 43.7 (0.60) | 55% | 320.81 (16.66) | 10.35 (.1529) |
|  | **1,417** | F | 43.3 (0.59) | 59% | 159.53 (11.56) | 11.48 (.1571) |
|  | **2,745** | T | 45.5 (0.42) | 57% | 237.57 (10.18) | 10.93 (.1105) |
| **Los Santos** | **1,087** | M | 45.8 (0.66) | 54% | 308.48 (13.21) | 10.31 (.1730) |
|  | **1,122** | F | 46.8 (0.67) | 61% | 159.81 (12.85) | 11.22 (.1747) |
|  | **2,209** | T | 46.3 (0.47) | 58% | 231.90 (9.38) | 10.78 (.1234) |
| **Veraguas** | **1,827** | M | 42.5 (0.55) | 41% | 221.56 (12.99) | 9.75 (.1460) |
|  | **1,716** | F | 42.5 (0.56) | 48% | 112.99 (7.42) | 10.19 (.1599) |
|  | **3,543** | T | 42.5 (0.39) | 45% | 167.37 (7.56) | 9.97 (.1083) |
| **Panama** | **3,287** | M | 39.3 (0.36) | 70% | 651.51 (27.13) | 13.47 (.0986) |
|  | **3,610** | F | 41.0 (0.35) | 68% | 368.69 (19.31) | 13.75 (.0933) |
|  | **6,897** | T | 40.2 (0.25) | 69% | 499.88 (16.43) | 13.62 (.0678) |
| **San Miguelito** | **1,632** | M | 40.2 (0.51) | 70% | 635.89 (29.35) | 13.34 (.1400) |
|  | **1,700** | F | 41.2 (0.51) | 68% | 332.61 (15.75) | 13.83 (.1378) |
|  | **3,332** | T | 40.8 (0.36) | 69% | 475.57 (16.47) | 13.60 (.0984) |
| **West Panama** | **1,894** | M | 39.7 (0.48) | 62% | 448.71 (15.53) | 12.01 (.1107) |
|  | **1,970** | F | 39.5 (0.47) | 61% | 233.85 (10.44) | 12.72 (.1193) |
|  | **3,864** | T | 39.6 (0.33) | 61% | 337.56 (9.47) | 12.38 (.0819) |
| **East Panama** | **1,404** | M | 41.2 (0.59) | 44% | 308.02 (14.31) | 9.12 (.1329) |
|  | **1,238** | F | 39.9 (0.59) | 40% | 109.34 (9.25) | 9.80 (.1659) |
|  | **2,642** | T | 40.6 (0.42) | 42% | 213.93 (9.03) | 9.44 (.1055) |
| **Kuna Yala** | **574** | M | 41.0 (1.11) | 14% | 141.81 (18.66) | 7.56 (.2907) |
|  | **657** | F | 40.1 (0.91) | 11% | 59.01 (12.71) | 5.53 (.2491) |
|  | **1,231** | T | 40.5 (0.71) | 13% | 94.78 (10.93) | 6.41 (.1926) |
| **Emberá** | **311** | M | 37.7 (1.36) | 8% | 140.02 (20.91) | 7.96 (.3567) |
|  | **287** | F | 38.8 (1.47) | 7% | 32.01 (7.80) | 5.57 (.3452) |
|  | **598** | T | 38.2 (1.00) | 8% | 88.57 (11.91) | 6.82 (.2575) |
| **Ngobe Bugle** | **1,090** | M | 36.4 (0.82) | 8% | 51.05 (5.41) | 6.54 (.2099) |
|  | **1,098** | F | 34.7 (0.70) | 6% | 27.37 (4.87) | 5.32 (.2114) |
|  | **2,188** | T | 35.5 (0.53) | 7% | 38.63 (3.63) | 5.90 (.1502) |
| **2013** | | | | | | |
| **Province/Region** | **n =** | **Sex** | **Age mean (SD)** | **With SS coverage** | **Income mean (SD)** | **Years of education mean (SD)** |
| **Bocas del Toro** | **1,216** | M | 37.2 (0.51) | 57% | 290.81 (13.79) | 9.62 (.1380) |
|  |  | F | 35.0 (0.46) | 48% | 148.35 (11.67) | 10.10 (1592) |
|  |  | T | 36.1 (0.35) | 52% | 217.93 (9.136) | 9.87 (.1058) |
| **Coclé** |  | M | 41.3 (0.62) | 47% | 279.85 (16.07) | 10.24 (.1356) |
|  |  | F | 42.2 (0.62) | 51% | 138.42 (12.38) | 11.18 (.1494) |
|  |  | T | 41.7 (0.44) | 49% | 208.74 (10.27) | 10.71 (.1494) |
| **Colón** |  | M | 37.1 (0.59) | 64% | 439.13 (15.56) | 11.76 (.1320) |
|  |  | F | 38.4 (0.59) | 55% | 191.40 (11.56) | 12.40 (.1504) |
|  |  | T | 37.8 (0.42) | 60% | 314.93 (10.30) | 12.08 (.1002) |
| **Chiriquí** | **1,241** | M | 41.8 (0.57) | 50% | 325.83 (16.40) | 10.88 (.1435) |
|  |  | F | 42.5 (0.55) | 59% | 153.66 (9.348) | 11.59 (.1513) |
|  |  | T | 42.2 (0.40) | 55% | 238.18 (9.499) | 11.24 (.1046) |
| **Darién** |  | M | 40.8 (0.70) | 17% | 272.47 (14.73) | 8.22 (.1775) |
|  |  | F | 37.8 (0.70) | 24% | 94.44 (9.56) | 8.78 (.2090) |
|  |  | T | 39.4 (0.50) | 20% | 189.76 (9.29) | 8.49 (.1359) |
| **Herrera** |  | M | 44.2 (0.62) | 52% | 384.06 (22.78) | 10.49 (.1567) |
|  |  | F | 43.9 (0.61) | 58% | 171.14 (11.46) | 11.43 (.1624) |
|  |  | T | 44.1 (0.43) | 55% | 273.06 (12.66) | 10.98 (.1136) |
| **Los Santos** |  | M | 46.2 (0.70) | 58% | 358.07 (23.01) | 10.57 (.1714) |
|  |  | F | 47.4 (0.69) | 62% | 158.12 (11.34) | 11.29 (.1811) |
|  |  | T | 46.8 (0.49) | 60% | 256.38 (12.94) | 10.93 (.1251) |
| **Veraguas** |  | M | 42.9 (0.55) | 42% | 236.28 (11.63) | 9.81 (.1452) |
|  |  | F | 43.1 (0.60) | 49% | 130.24 (8.720) | 10.33 (.1660) |
|  |  | T | 43.0 (0.41) | 45% | 184.66 (7.416) | 10.07 (.1100) |
| **Panama** |  | M | 39.0 (0.40) | 68% | 684.01 (32.28) | 13.42 (.1115) |
|  |  | F | 40.9 (0.41) | 65% | 358.40 (20.31) | 13.69 (.1027) |
|  |  | T | 40.0 (0.29) | 67% | 514.08 (19.05) | 13.56 (.0756) |
| **San Miguelito** |  | M | 40.8 (0.57) | 70% | 635.86 (36.80) | 13.71 (.1540) |
|  |  | F | 41.5 (0.53) | 71% | 367.81 (21.14) | 14.02 (.1468 |
|  |  | T | 41.1 (0.39) | 70% | 491.86 (20.93) | 13.87 (.1062) |
| **West Panama** |  | M | 39.5 (0.51) | 65% | 489.59 (16.75) | 12.22 (.1127) |
|  |  | F | 40.0 (0.48) | 63% | 238.93 (10.08) | 12.73 (.1222) |
|  |  | T | 39.8 (0.35) | 63% | 361.07 (10.00) | 12.48 (.0834) |
| **East Panama** |  | M | 41.2 (0.63) | 41% | 369.69 (18.36) | 9.28 (.1398) |
|  |  | F | 39.6 (0.60) | 39% | 119.00 (7.93) | 9.98 (.1587) |
|  |  | T | 40.4 (0.44) | 40% | 249.95 (10.72) | 9.61 (.1057) |
| **Kuna Yala** |  | M | 40.5 (1.22) | 11% | 97.35 (14.15) | 7.94 (.2934) |
|  |  | F | 40.2 (0.97) | 9% | 39.41 (6.293) | 5.61 (.2732) |
|  |  | T | 40.3 (0.76) | 10% | 65.30 (7.282) | 6.65 (.2048) |
| **Emberá** |  | M | 38.2 (1.32) | 15% | 173.66 (15.72) | 7.80 (.3253) |
|  |  | F | 38.0 (1.37) | 6% | 62.52 (16.26) | 6.11 (.3665) |
|  |  | T | 39.1 (0.95) | 11% | 122.08 (11.61) | 7.02 (.2471) |
| **Ngabe Bugle** |  | M | 38.0 (0.91) | 7% | 45.31 (5.83) | 6.05 (.2271) |
|  |  | F | 35.8 (0.76) | 4% | 13.52 (2.52) | 4.43 (.2013) |
|  |  | T | 36.8 (0.59) | 6% | 27.80 (2.99) | 5.15 (.1527) |
| **2014** | | | | | | |
| **Province/Region** |  | **Sex** | **Age mean (SD)** | **With SS coverage** | **Income mean (SD)** | **Years of education mean (SD)** |
| **Bocas del Toro** |  | M | 36.9 (0.50) | 59% | 308.91 (13.27) | 9.91 (.1423) |
|  |  | F | 35.0 (0.46) | 49% | 129.04 (7.674) | 10.11 (.1638) |
|  |  | T | 36.0 (0.34) | 53% | 218.53 (7.865) | 10.02 (.1085) |
| **Coclé** |  | M | 43.0 (0.62) | 47% | 263.10 (11.56) | 10.13(.1299) |
|  |  | F | 42.7 (0.60) | 51% | 125.21 (8.877) | 11.09 (.1418) |
|  |  | T | 42.9 (0.43) | 49% | 193.38 (7.424) | 10.62 (.0968) |
| **Colón** |  | M | 38.2 (0.53) | 57% | 471.97 (22.40) | 12.02 (.1256) |
|  |  | F | 38.4 (0.53) | 52% | 214.55 (11.66) | 12.59 (.1368) |
|  |  | T | 38.3 (0.38) | 55% | 341.81 (12.87) | 12.31 (.0931) |
| **Chiriquí** |  | M | 42.5 (0.55) | 48% | 404.92 (38.93) | 11.33 (.1413) |
|  |  | F | 43.3 (0.56) | 57% | 163.02 (9.497) | 11.85 (.1486) |
|  |  | T | 42.9 (0.39) | 53% | 280.19 (19.64) | 11.60 (.1029) |
| **Darién** |  | M | 39.9 (0.69) | 22% | 322.90 (26.15) | 8.71 (.1757) |
|  |  | F | 37.1 (0.66) | 22% | 117.74 (10.37) | 9.41 (.2136) |
|  |  | T | 38.6 (0.49) | 22% | 226.76 (14.96) | 9.04 (.1372) |
| **Herrera** |  | M | 44.9 (0.62) | 52% | 340.70 (15.29) | 10.31 (.1531) |
|  |  | F | 44.9 (0.60) | 62% | 198.76 (13.63) | 11.47 (.1610) |
|  |  | T | 44.9 (0.43) | 57% | 366.71 (10.32) | 10.91 (.1121) |
| **Los Santos** |  | M | 45.8 (0.67) | 55% | 361.71 (21.58) | 10.88 (.1658) |
|  |  | F | 47.3 (0.66) | 64% | 170.00 (13.80) | 11.51 (.1701) |
|  |  | T | 46.5 (0.47) | 59% | 266.12 (13.02) | 11.19 (.1189) |
| **Veraguas** |  | M | 43.0 (0.53) | 40% | 269.32 (11.31) | 10.01 (.1410) |
|  |  | F | 43.1 (0.56) | 48% | 137.04 (8.988) | 10.42 (.1658) |
|  |  | T | 43.0 (0.39) | 44% | 205.11 (7.387) | 10.21 (.1084) |
| **Panama** |  | M | 39.6 (0.39) | 67% | 657.43 (22.50) | 13.44 (.1069) |
|  |  | F | 40.6 (0.37) | 64% | 386.29 (16.07) | 13.81 (.1024) |
|  |  | T | 40.1 (0.27) | 65% | 512.09 (13.72) | 13.64 (.0740) |
| **San Miguelito** |  | M | 40.8 (0.57) | 71% | 634.78 (31.12) | 13.66 (.1354) |
|  |  | F | 41.4 (0.52) | 72% | 392.37 (19.37) | 13.83 (.1340) |
|  |  | T | 41.2 (0.38) | 71% | 502.36 (17.94) | 13.75 (.0956) |
| **West Panama** |  | M | 39.8 (0.49) | 62% | 539.43 (23.30) | 12.15 (.1148) |
|  |  | F | 40.6 (0.49) | 62% | 269.12 (11.38) | 12.80 (.1188) |
|  |  | T | 40.2 (0.34) | 62% | 403.01 (13.19) | 12.47 (.0828) |
| **East Panama** |  | M | 40.8 (0.62) | 37% | 331.05 (14.55) | 9.09 (.1360) |
|  |  | F | 39.8 (0.61) | 40% | 148.20 (9.40) | 10.11 (.1605) |
|  |  | T | 40.3 (0.44) | 39% | 242.38 (9.01) | 9.59 (.1055) |
| **Kuna Yala** |  | M | 40.6 (1.23) | 10% | 112.51 (13.59) | 7.10 (.3094) |
|  |  | F | 39.8 (1.02) | 7% | 44.73 (9.504) | 5.33 (.2910) |
|  |  | T | 40.2 (0.79) | 8% | 75.05 (8.120) | 6.13 (.2147) |
| **Emberá** |  | M | 40.3 (1.51) | 11% | 144.20 (15.78) | 8.034 (.3579) |
|  |  | F | 39.4 (1.60) | 7% | 105.48 (73.16) | 6.274 (.4146) |
|  |  | T | 39.8 (1.10) | 9% | 126.00 (35.35) | 7.206 (.2756) |
| **Ngabe Bugle** |  | M | 36.4 (0.92) | 8% | 53.12 (7.981) | 6.73 (.2363) |
|  |  | F | 35.3 (0.75) | 3% | 10.90 (2.479) | 4.79 (.2069) |
|  |  | T | 35.8 (0.59) | 5% | 29.65 (3.867) | 5.65 (.1587) |
| **2015** | | | | | | |
| **Province/Region** |  | **Sex** | **Age mean (SD)** | **With SS coverage** | **Income mean (SD)** | **Years of education mean (SD)** |
|  |  |  | |  |  |  |
| **Bocas del Toro** |  | M | 37.1 (0.53) | 58% | 325.28 (14.94) | 9.79 (.1515) |
|  |  | F | 35.6 (0.49) | 49% | 151.81 (9.41) | 10.10 (.1753) |
|  |  | T | 36.3 (0.36) | 53% | 238.22 (9.01) | 9.94 (.1159) |
| **Coclé** |  | M | 43.1 (0.62) | 50% | 317.10 (19.07) | 10.48 (.1314) |
|  |  | F | 43.8 (0.61) | 52% | 155.59 (11.19) | 11.17 (.1443) |
|  |  | T | 43.45 (0.43) | 51% | 234.42 (11.07) | 10.83 (.0980) |
| **Colón** |  | M | 39.7 (0.54) | 57% | 477.51 (20.79) | 12.26 (.1280) |
|  |  | F | 39.1 (0.52) | 57% | 221.35 (11.79) | 12.99 (.1301) |
|  |  | T | 39.4 (0.37) | 57% | 343.95 (12.06) | 12.64 (.0917) |
| **Chiriquí** |  | M | 42.6 (0.55) | 52% | 410.96 (26.73) | 11.50 (.1437) |
|  |  | F | 43.1 (0.53) | 56% | 205.52 (14.48) | 12.27 (.1502) |
|  |  | T | 42.8 (0.38) | 54% | 306.19 (15.18) | 11.89 (.1043) |
| **Darién** |  | M | 39.5 (0.69) | 20% | 269.20 (13.74) | 8.92 (.1684) |
|  |  | F | 37.4 (0.67) | 22% | 114.21 (10.39) | 9.47 (.2025) |
|  |  | T | 38.5 (0.48) | 21% | 195.91 (8.97) | 9.18 (.1308) |
| **Herrera** |  | M | 45.5 (0.64) | 52% | 359.68 (18.36) | 10.57 (.1577) |
|  |  | F | 45.2 (0.62) | 65% | 196.99 (13.10) | 11.74 (.1631) |
|  |  | T | 45.3 (0.45) | 59% | 274.76 (11.27) | 11.18 (.1144) |
| **Los Santos** |  | M | 46.2 (0.65) | 55% | 370.15 (16.59) | 10.81 (.1624) |
|  |  | F | 47.3 (0.67) | 62% | 206.32 (14.71) | 11.86 (.1755) |
|  |  | T | 46.8 (0.47) | 59% | 287.62 (11.25) | 11.34 (.1202) |
| **Veraguas** |  | M | 43.2 (0.55) | 41% | 274.525 (11.87) | 10.29 (.1469) |
|  |  | F | 43.0 (0.57) | 51% | 184.16 (13.57) | 10.95 (.1655) |
|  |  | T | 43.1 (0.40) | 46% | 230.12 (9.04) | 10.62 (.1106) |
| **Panama** |  | M | 39.7 (0.42) | 65% | 746.96 (34.54) | 13.39 (.1028) |
|  |  | F | 41.1 (0.39) | 64% | 418.49 (26.79) | 13.81 (.1010) |
|  |  | T | 40.4 (0.29) | 64% | 575.14 (21.85) | 13.61 (.0722) |
| **San Miguelito** |  | M | 39.8 (0.52) | 67% | 699.06 (34.69) | 13.62 (.1360) |
|  |  | F | 42.2 (0.55) | 66% | 405.39 (25.16) | 13.69 (.1365) |
|  |  | T | 41.0 (0.38) | 67% | 541.77 (21.31) | 13.66 (.0966) |
| **West Panama** |  | M | 40.3 (0.51) | 64% | 525.25 (19.57) | 12.14 (.1204) |
|  |  | F | 41.1 (0.52) | 64% | 294.43 (13.67) | 12.76 (.1299) |
|  |  | T | 40.7 (0.36) | 64% | 409.01 (12.14) | 12.46 (.0888) |
| **East Panama** |  | M | 39.7 (0.59) | 38% | 368.31 (18.41) | 9.53 (.1339) |
|  |  | F | 39.6 (0.58) | 41% | 156.31 (11.67) | 10.47 (.1578) |
|  |  | T | 39.6 (0.42) | 40% | 267.23 (11.42) | 9.97 (.1032) |
| **Kuna Yala** |  | M | 40.4 (1.22) | 10% | 115.73 (13.64) | 7.36 (.2909) |
|  |  | F | 39.1 (0.95) | 8% | 57.62 (9.82) | 5.37 (.2814) |
|  |  | T | 39.7 (0.76) | 9% | 83.26 (8.20) | 6.25 (.2064) |
| **Emberá** |  | M | 40.3 (1.6) | 18% | 176.70 (22.43) | 8.49 (.3484) |
|  |  | F | 42.8 (1.78) | 10% | 42.44 (9.66) | 5.81 (.4407) |
|  |  | T | 41.4 (1.20) | 15% | 118.31 (13.89) | 7.32 (.2860) |
| **Ngabe Bugle** |  | M | 35.7 (0.91) | 7% | 84.15 (11.81) | 7.09 (.2479) |
|  |  | F | 35.9 (0.80) | 6% | 20.56 (4.78) | 5.26 (.2195) |
|  |  | T | 35.9 (0.60) | 7% | 49.36 (6.04) | 6.09 (.1671) |
| **2016** | | | | | | |
| **Province/Region** |  | **Sex** | **Age mean (SD)** | **With SS coverage** | **Income mean (SD)** | **Years of education mean (SD)** |
| **Bocas del Toro** |  | M | 37.9 (0.92) | 49% | 274.09 (16.02) | 9.71 (.2264) |
|  |  | F | 35.6 (0.82) | 45% | 155.69 (12.88) | 9.73 (.2453) |
|  |  | T | 36.7 (0.61) | 47% | 212.97 (10.26) | 9.73 (.1674) |
| **Coclé** |  | M | 42.8 (0.60) | 49% | 345.85 (17.25) | 10.64 (.1314) |
|  |  | F | 44.6 (0.61) | 55% | 170.48 (13.04) | 11.30 (.1453) |
|  |  | T | 43.7 (0.43) | 52% | 257.68 (10.95) | 10.97 (.0983) |
| **Colón** |  | M | 37.5 (0.50) | 58% | 481.61 (18.38) | 12.38 (.1123) |
|  |  | F | 39.5 (0.51) | 54% | 233.19 (12.598) | 13.06 (.1239) |
|  |  | T | 38.5 (0.36) | 56% | 353.49 (11.37) | 12.73 (.0841) |
| **Chiriquí** |  | M | 42.6 (0.57) | 51% | 385.11 (15.53) | 11.52 (.1419) |
|  |  | F | 42.3 (0.52) | 54% | 204.36 (16.13) | 12.45 (.1416) |
|  |  | T | 42.4 (0.39) | 53% | 290.18 (11.39) | 12.01 (.1007) |
| **Darién** |  | M | 40.9 (1.24) | 16% | 224.95 (16.56) | 7.90 (.2803) |
|  |  | F | 41.4 (1.32) | 18% | 111.36 (13.52) | 7.85 (.3861) |
|  |  | T | 41.1 (0.91) | 17% | 170.69 (10.72) | 7.88 (.2355) |
| **Herrera** |  | M | 45.1 (0.64) | 51% | 350.87 (15.10) | 10.69 (.1607) |
|  |  | F | 45.1 (0.63) | 64% | 205.80 (14.31) | 12.01 (.1652) |
|  |  | T | 45.1 (0.45) | 58% | 276.36 (10.51) | 11.37 (.1162) |
| **Los Santos** |  | M | 46.4 (0.68) | 56% | 398.74 (18.28) | 10.87 (.1677) |
|  |  | F | 47.0 (0.67) | 64% | 229.89 (14.59) | 12.06 (.1760) |
|  |  | T | 46.7 (0.48) | 60% | 312.42 (11.81) | 11.48 (.1225) |
| **Veraguas** |  | M | 43.5 (0.56) | 45% | 308.89 (15.56) | 10.47 (.1458) |
|  |  | F | 43.4 (0.57) | 55% | 199.37 (13.20) | 11.22 (.16.77) |
|  |  | T | 43.4 (0.40) | 50% | 253.76 (10.25) | 10.85 (.1114) |
| **Panama** |  | M | 39.5 (0.40) | 66% | 704.37 (23.52) | 13.55 (.0968) |
|  |  | F | 41.4 (0.38) | 66% | 416.24 (17.86) | 13.84 (.1021) |
|  |  | T | 40.5 (0.28) | 66% | 555.93 (14.86) | 13.70 (.0705) |
| **San Miguelito** |  | M | 40.0 (0.51) | 67% | 788.95 (42.48) | 13.84 (.1263) |
|  |  | F | 42.8 (0.53) | 68% | 486.73 (27.34) | 13.83 (.1291) |
|  |  | T | 41.4 (0.35) | 68% | 632.97 (25.22) | 13.84 (.0904) |
| **West Panama** |  | M | 40.4 (0.51) | 61% | 572.53 (22.56) | 12.13 (.1197) |
|  |  | F | 41.1 (0.50) | 62% | 305.89 (14.00) | 12.83 (.1231) |
|  |  | T | 40.8 (0.36) | 61% | 434.66 (13.34) | 12.49 (.0862) |
| **East Panama** |  | M | 37.2 (1.32) | 33% | 280.46 (21.56) | 7.98 (.5139) |
|  |  | F | 36.3 (1.59) | 31% | 109.22 (11.92) | 7.77 (.5427) |
|  |  | T | 36.7 (1.04) | 32% | 189.94 (11.94) | 7.86 (.3758) |
| **Kuna Yala** |  | M | 40.7 (1.20) | 13% | 145.43 (14.90) | 7.79 (.2933) |
|  |  | F | 40.6 (1.04) | 9% | 41.45 (7.170) | 5.30 (.2606) |
|  |  | T | 40.7 (0.79) | 11% | 87.62 (7.997) | 6.40 (.2014) |
| **Emberá** |  | M | 40.0 (1.79) | 16% | 165.70 (21.62) | 7.96 (.4090) |
|  |  | F | 37.7 (1.76) | 10% | 57.40 (14.06) | 6.67 (.4686) |
|  |  | T | 39.0 (1.27) | 13% | 118.74 (14.08) | 7.40 (.3101) |
| **Ngabe Bugle** |  | M | 36.0 (0.95) | 11% | 86.62 (11.49) | 7.21 (.2439) |
|  |  | F | 36.2 (0.79) | 6% | 31.05 (5.823) | 5.77 (.2233) |
|  |  | T | 36.1 (0.61) | 8% | 55.81 (6.113) | 6.41 (.1661) |
