# Supplementary material for: Social determinants and cardiovascular disease mortality in Panama, 2012–2016
Source: BMC Public Health. 2019 Feb 15;19:199. doi: 10.1186/s12889-019-6508-8 (PMC6377740; doi:10.1186/s12889-019-6508-8)
Supplement: Supplementary file 2 — Variation in socioeconomic variables and cardiovascular disease mortality rates. The table shows the variations in all the socioeconomic variables and IHD/stroke mortality in men and women throughout the provinces and regions (between) and throughout the years (within variation). (DOCX 16 kb) [file 12889_2019_6508_MOESM2_ESM.docx]

**Additional file 2. Variation in socioeconomic variables and cardiovascular disease mortality rates.**

| **Socioeconomic Variable** | **Variation** | **Mean** | **Standard Deviation** | **Min** | **Max** |
| --- | --- | --- | --- | --- | --- |
| **IHD mortality rate men** | overall | 79.55 | 28.59595 | 33.87 | 151.91 |
|  | between |  | 21.53519 | 43.64 | 119.174 |
|  | within |  | 19.63174 | 30.28483 | 144.4748 |
| **IHD mortality rate women** | overall | 38.92 | 18.03918 | 0 | 94.06 |
|  | between |  | 12.12124 | 24.46 | 56.422 |
|  | within |  | 13.72766 | 8.421167 | 78.90117 |
| **Stroke mortality rate men** | overall | 61.78 | 15.38451 | 22.2 | 105.22 |
|  | between |  | 8.052231 | 49.744 | 72.84 |
|  | within |  | 13.27556 | 28.714 | 104.03 |
| **Stroke mortality rate women** | overall | 38.22 | 14.83007 | 9.58 | 85.41 |
|  | between |  | 9.91304 | 13.65 | 51.804 |
|  | within |  | 11.32806 | 9.303167 | 79.14717 |
| **Access to social security** | overall | 0.52 | 0.1360608 | 0.1596 | 0.7069 |
|  | between |  | 0.1390175 | 0.18932 | 0.69012 |
|  | within |  | 0.0222911 | 0.44711 | 0.56655 |
| **Monthly income** | overall | 405.13 | 149.8638 | 221.56 | 788.95 |
|  | between |  | 150.5636 | 262.115 | 688.856 |
|  | within |  | 36.42344 | 331.5408 | 515.1708 |
| **Years of education** | overall | 10.93 | 1.605856 | 7.9 | 13.84 |
|  | between |  | 1.641751 | 8.366 | 13.634 |
|  | within |  | 0.2572172 | 9.913833 | 11.48783 |
